# Identification of key genes and immune infiltration in osteoarthritis through analysis of zinc metabolism-related genes

**DOI:** 10.1186/s12891-024-07347-8

**Published:** 2024-03-21

**Authors:** Xiaoxuan You, Yanbo Ye, Shufeng Lin, Zefeng Zhang, Huiyang Guo, Hui Ye

**Affiliations:** 1 Department of Orthopedics, The Second Affiliated Hospital of Fujian Medical University, 34 Zhongshan North Road, Licheng District, Quanzhou, 362000 Fujian China; 2 Suzhou University Medical Department, Suzhou, 215000 Jiangsu China

**Keywords:** Zinc metabolism-related genes, Osteoarthritis, Diagnosis, Hub genes, Protein-protein interaction

## Abstract

**Background:**

Osteoarthritis (OA) represents a prominent etiology of considerable pain and disability, and conventional imaging methods lack sensitivity in diagnosing certain types of OA. Therefore, there is a need to identify highly sensitive and efficient biomarkers for OA diagnosis. Zinc ions feature in the pathogenesis of OA. This work aimed to investugate the role of zinc metabolism-related genes (ZMRGs) in OA and the diagnostic characteristics of key genes.

**Methods:**

We obtained datasets GSE169077 and GSE55235 from the Gene Expression Omnibus (GEO) and obtained ZMRGs from MSigDB. Differential expression analysis was conducted on the GSE169077 dataset using the limma R package to identify differentially expressed genes (DEGs), and the intersection of DEGs and ZMRGs yielded zinc metabolism differential expression-related genes (ZMRGs-DEGs). The clusterProfiler R package was employed for Gene Ontology (GO) and Kyoto Encyclopedia of Genes and Genomes (KEGG) enrichment analyses of ZMRGs-DEGs. Potential small molecule drugs were predicted using the CMap database, and immune cell infiltration and function in OA individuals were analyzed using the ssGSEA method. Protein-protein interaction (PPI) networks were constructed to detect Hub genes among ZMRGs-DEGs. Hub gene expression levels were analyzed in the GSE169077 and GSE55235 datasets, and their diagnostic characteristics were assessed using receiver operating characteristic (ROC) curves. The gene-miRNA interaction network of Hub genes was explored using the gene-miRNA interaction network website.

**Results:**

We identified 842 DEGs in the GSE169077 dataset, and their intersection with ZMRGs resulted in 46 ZMRGs-DEGs. ZMRGs-DEGs were primarily enriched in functions such as collagen catabolic processes, extracellular matrix organization, metallopeptidase activity, and pathways like the IL-17 signaling pathway, Nitrogen metabolism, and Relaxin signaling pathway. Ten potential small-molecule drugs were predicted using the CMap database. OA patients exhibited distinct immune cell abundance and function compared to healthy individuals. We identified 4 Hub genes (MMP2, MMP3, MMP9, MMP13) through the PPI network, which were highly expressed in OA and demonstrated good diagnostic performance. Furthermore, two closely related miRNAs for each of the 4 Hub genes were identified.

**Conclusion:**

4 Hub genes were identified as potential diagnostic biomarkers and therapeutic targets for OA.

**Supplementary Information:**

The online version contains supplementary material available at 10.1186/s12891-024-07347-8.

## Introduction

Osteoarthritis (OA) is a chronic degenerative joint disorder characterized by synovial inflammation, cartilage degeneration, and reduced joint function [1]. Patients typically manifest symptoms including swelling, pain, and stiffness, which adversely affect their quality of life [2]. In recent decades, the incidence of OA has been on the rise due to aging populations and obesity [3]. According to recent research, by 2030, China is expected to have approximately 400 million cases of OA [4]. Clinical imaging findings often do not align with the symptoms experienced by patients [5]. Hence, a pressing need exists for the development of exceptionally sensitive and efficient biomarkers for the diagnosis of OA, as well as to facilitate the exploration of OA pathogenesis in the context of personalized treatment.

The potential involvement of zinc ions in the pathogenesis of OA has garnered increasing attention. Clinical studies have demonstrated a notable elevation in serum zinc levels in OA patients [6]. Research has also unveiled a correlation between daily zinc intake and the incidence of OA [7], and genetic predictions have linked zinc cycling levels to OA development [8]. The connection between zinc ions and OA pathogenesis is widely understood as zinc ions being essential structural components of matrix-degrading enzymes, necessary for their maturation and activation [9]. In human cell lines and mouse models, zinc influx into chondrocytes upregulates zinc-activated transcription factor MTF1, inducing the expression of matrix-degrading enzymes, leading to cartilage destruction and OA development [9]. Currently, the use of zinc chelators targeting aggrecanases has proven to be effective in OA treatment [10]. Therefore, further analysis of zinc metabolism-related genes (ZMRGs) associated with OA is warranted, as it holds the promise of offering potential therapeutic targets.

This work garnered data from OA cases and healthy controls from the Gene Expression Omnibus (GEO) database, identified differentially expressed genes (DEGs), and then intersected DEGs with ZMRGs to obtain zinc metabolism differential expression-related genes (ZMRGs-DEGs). The functions of ZMRGs-DEGs were dissected using Gene Ontology (GO) and Kyoto Encyclopedia of Genes and Genomes (KEGG) analyses. Potential small molecule drugs were predicted using the CMap database, and the response to immunotherapy and immune infiltration in OA patients was analyzed using the ssGSEA method. Protein-protein interaction (PPI) networks were built to identify Hub genes among ZMRGs-DEGs. Subsequently, the expression of Hub genes was analyzed in GSE169077 and GSE55235 datasets, and their diagnostic characteristics were assessed using receiver operating characteristic (ROC) curves, to provide further insights into the zinc metabolism mechanism in OA and the diagnosis and treatment of OA.

## Materials and methods

### Data sources

**Fig. 1:**
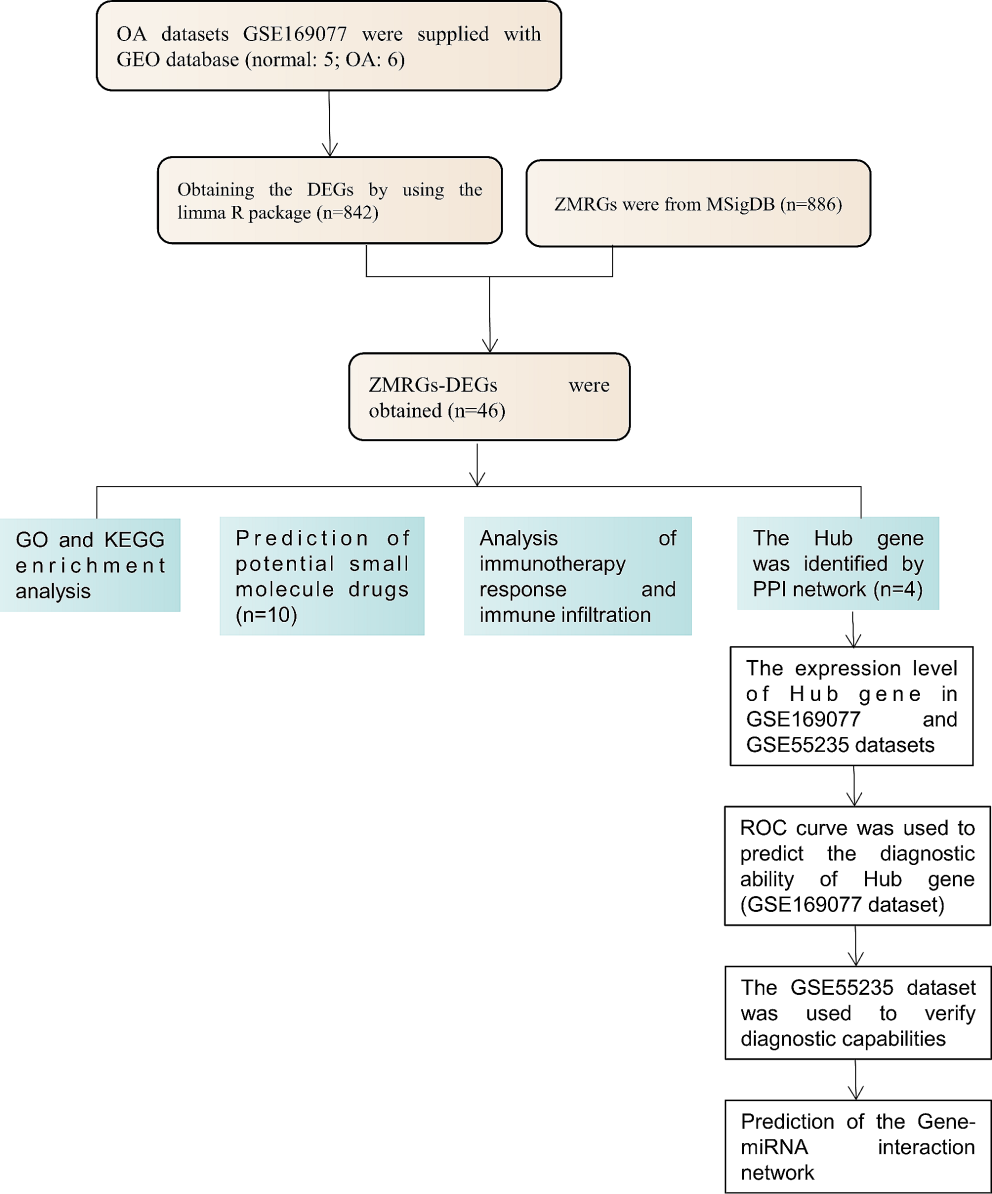
A workflow of this study

OA datasets GSE169077 and GSE55235 were supplied with the GEO database (https://www.ncbi.nlm.nih.gov/). GSE169077 consists of 5 normal samples and 6 OA samples, while GSE55235 contains 10 normal samples and 10 OA samples. ZMRGs were from MSigDB [11], resulting in 886 ZMRGs after removing duplicate genes (Supplementary Table 1). A brief workflow for this study is shown in Fig. 1.

### Analysis of DEGs

DEGs were identified in the GSE169077 dataset by using the limma R package, with selection criteria of *P*-value < 0.05 and |LogFC| > 1. The intersection of DEGs and ZMRGs yielded ZMRGs-DEGs.

### KEGG and GO enrichment analyses

Utilizing the clusterProfiler R package, we conducted GO analysis on ZMRGs-DEGs, including categories such as biological processes, molecular functions, and cellular components. Additionally, we performed a KEGG pathway enrichment analysis with a significance threshold of *P* < 0.05.

### Prediction of potential small molecule drugs

The Connectivity Map (CMap) database (https://portals.broadinstitute.org/cmap/) is an indispensable resource in pharmacogenomics that links diseases to drugs, where negative scores suggest potential benefits of a drug for disease treatment [12]. We input the 30 upregulated genes from ZMRGs-DEGs into the CMap database to obtain information on potential drugs and select the top 10 drugs for presentation.

### Analysis of immunotherapy response and immune infiltration

Using the ssGSEA method, we assessed the levels of immune-related cells and immune-related functions in the disease group compared to the normal group. Additionally, the expression of immune checkpoint genes in the normal and disease groups was evaluated.

### Identification of hub genes among ZMRGs-DEGs

A PPI network was constructed for ZMRGs-DEGs utilizing the STRING database (https://string-db.org/), with a confidence score threshold > 0.4. Based on PPI network data obtained from the STRING database, the top 4 Hub genes were selected using the cytoHubba plugin in Cytoscape, employing the MCC algorithm.

### Validation of hub genes’ diagnostic capability

Expression data for Hub genes were extracted from the GSE169077 and GSE55235 datasets and visualized using the ggplot2 R package. The predictive capability of Hub genes was assessed by generating ROC curves and calculating the area under the curve (AUC) using the pROC R package on the GSE169077 dataset and subsequently validated on the GSE55235 dataset.

### Prediction of the gene-miRNA interaction network

An interaction network between genes, miRNAs, and Hub genes was created using the website (http://mirwalk.umm.uni-heidelberg.de/). Parameters used for this analysis included specified organism: Homo sapiens (Human) and collection ID type (Official gene symbols).

## Results

### Selection and functional enrichment of ZMRGs-DEGs

**Fig. 2:**
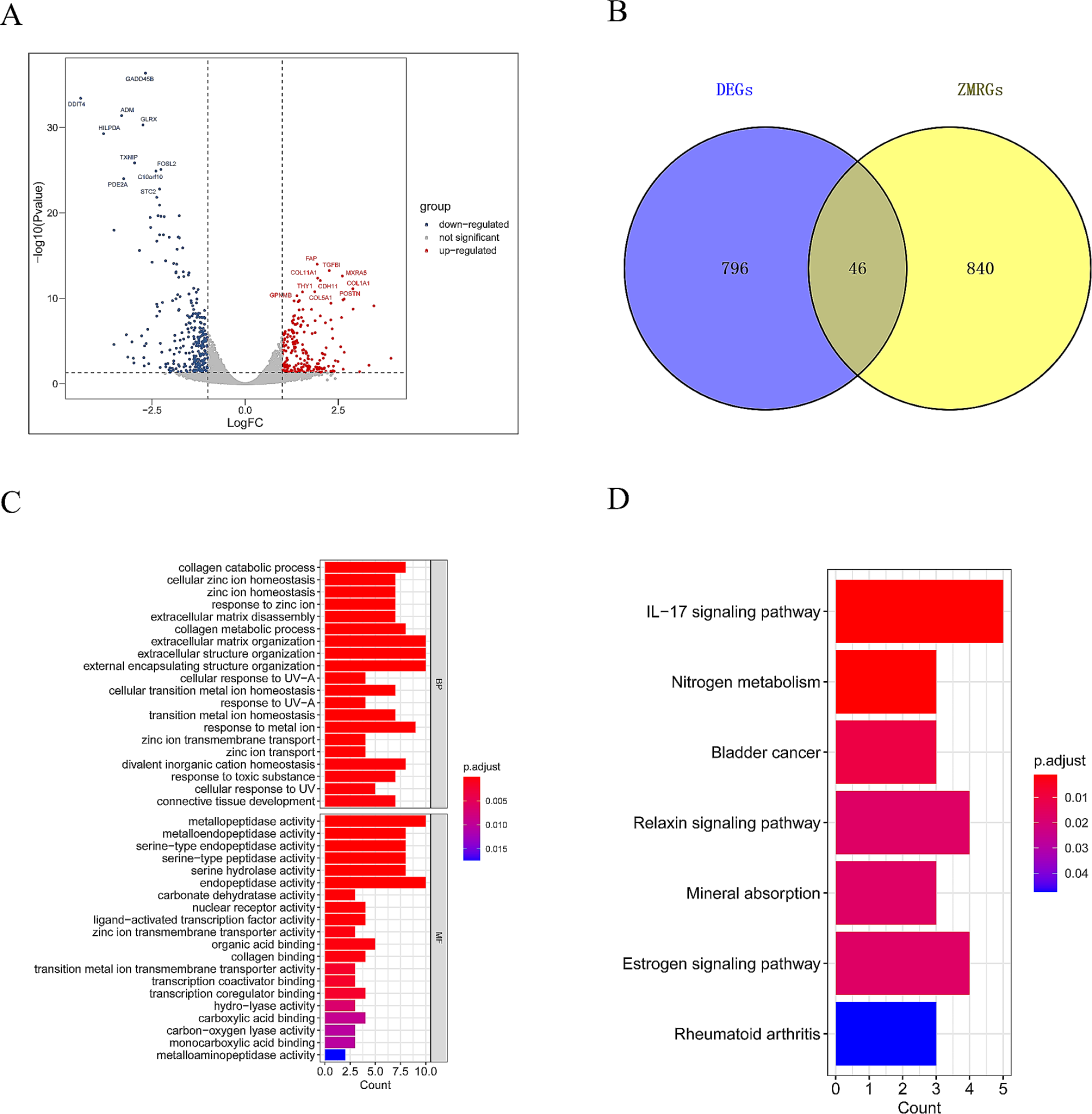
Acquisition of ZMRGs-DEGs and functional enrichment analysis based on the GEO database **(A)** DEGs between normal and OA samples in the GSE169077 dataset were analyzed and plotted as a volcano plot (*P* value < 0.05, |LogFC|> 1). **(B)** Intersection of DEGs and ZMRGs in the GSE169077 dataset. **(C-D)** ZMRGs-DEGs were analyzed using the clusterProfiler R package for GO functional enrichment analysis and KEGG pathway enrichment analysis

In the GSE169077 dataset, we identified a total of 842 DEGs based on the selection criteria of *P*-value < 0.05 and |LogFC| > 1. Among them, 402 genes were downregulated, and 440 genes were upregulated (Fig. 2A). The intersection of DEGs and ZMRGs yielded 46 ZMRGs-DEGs (Fig. 2B).

To explore the functions of ZMRGs-DEGs, we conducted GO and KEGG enrichment analyses using the clusterProfiler R package. The GO outcomes revealed that ZMRGs-DEGs were predominantly enriched in functions related to collagen catabolic processes, extracellular matrix organization, metallopeptidase activity, and more (Fig. 2C). Additionally, the KEGG outcomes indicated that ZMRGs-DEGs were primarily enriched in metabolic pathways like the IL-17 signaling pathway, Nitrogen metabolism, Relaxin signaling pathway, and others (Fig. 2D).

### Identification of potential small molecule drugs

We input the 30 upregulated ZMRGs-DEGs (Supplementary Table 2) into the CMap database to obtain information about potential drugs. As shown in Table 1, we identified 10 small molecule compounds that might be potential therapeutic drugs for OA, including pantoprazole, cholic-acid, AS-604,850, etomoxir, mupirocin, naftopidil, PP-3, deferiprone, SR-95,639 A, and KUC103898N.

**Table 1:** Top 10 small molecule compounds with the highest negative correlation scores identified from the CMap database

Name	ID	Description	Score
pantoprazole	BRD-A22380646	ATPase inhibitor	-99.89
cholic-acid	BRD-K43164539	Bile acid	-99.79
AS-604,850	BRD-K63915849	PI3K inhibitor Carnitine	-99.79
etomoxir	BRD-K77625572	Palmitoyltransferase inhibitor	-99.51
mupirocin	BRD-K15262564	Isoleucyl-tRNA synthetase inhibitor	-99.33
naftopidil	BRD-A01787639	Adrenergic receptor antagonist	-99.25
PP-3	BRD-K16977723	EGFR inhibitor	-99.19
deferiprone	BRD-K06878038	Chelating agent	-99.12
SR-95,639 A	BRD-K19309090	Acetylcholine receptor agonist	-99.09
KUC103898N	BRD-K34508425	-666	-99.08

### Prediction of immunotherapy response and immune infiltration analysis

**Fig. 3:**
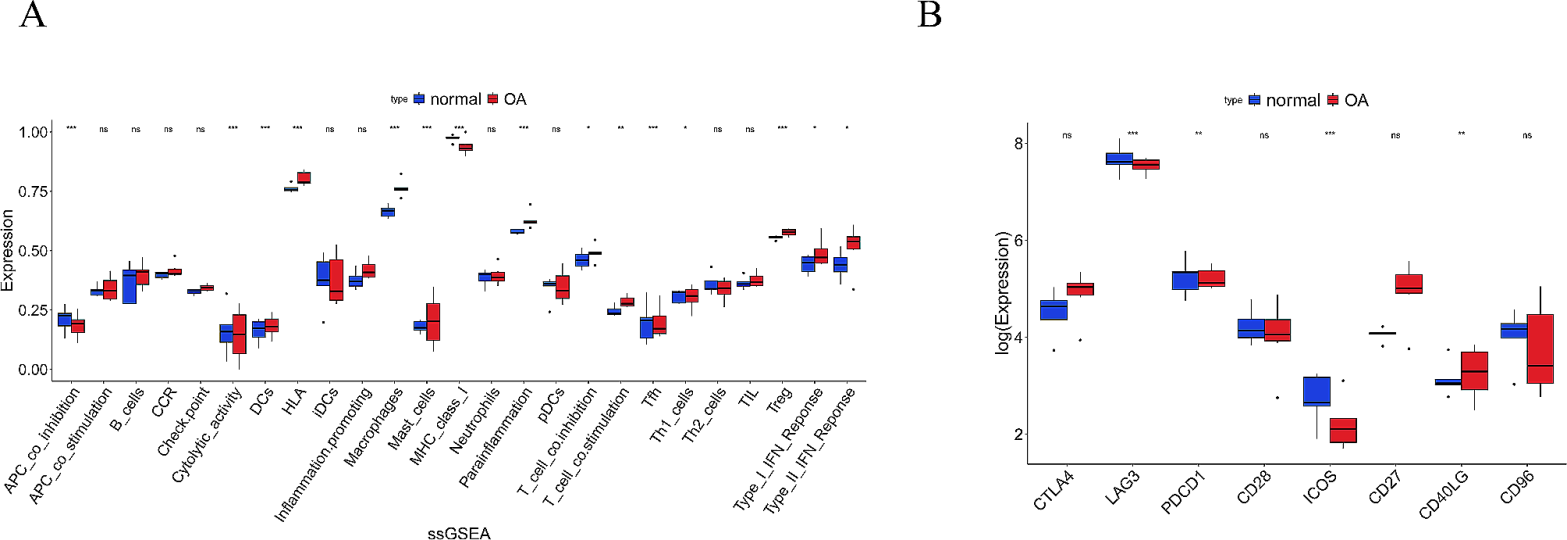
Immune infiltration levels and expression levels of immune checkpoint genes in OA patients and normal patients analyzed by ssGSEA. **(A)** ssGSEA analysis of immune cells and immune-related functions in OA patients and normal patients. **(B)** Expression level of immune checkpoint genes in OA patients and normal patients; * indicates *P* < 0.05; ** indicates *P* < 0.01; *** indicates *P* < 0.001

To explore the immunotherapy response and immune infiltration in OA, we utilized ssGSEA to assess the differences in immune-related cells and immune-related functions between OA patients and normal individuals. As the results revealed, in OA patients, the abundance of DCs, Macrophages, Mast Cells, and Treg cells significantly increased, while the abundance of Tfh and Th1_cells significantly decreased (Fig. 3A). Additionally, the abundance of immune-related functions in OA patients significantly decreased in APC_co_inhibition, Cytolytic_activity, and MHC_class_I, while it significantly increased in HLA, Parainflammation, and T_cell_co.inhibition, T_cell_co.stimulation, Type_I_IFN_Response, and Type_II_IFN_Response (Fig. 3A). We further assessed the expression of immune checkpoint genes in OA. The results showed that CD40LG was significantly upregulated in OA patients, while LAG3, PDCD1, and ICOS were significantly downregulated (Fig. 3B).

### Identification of hub genes among ZMRGs-DEGs

**Fig. 4:**
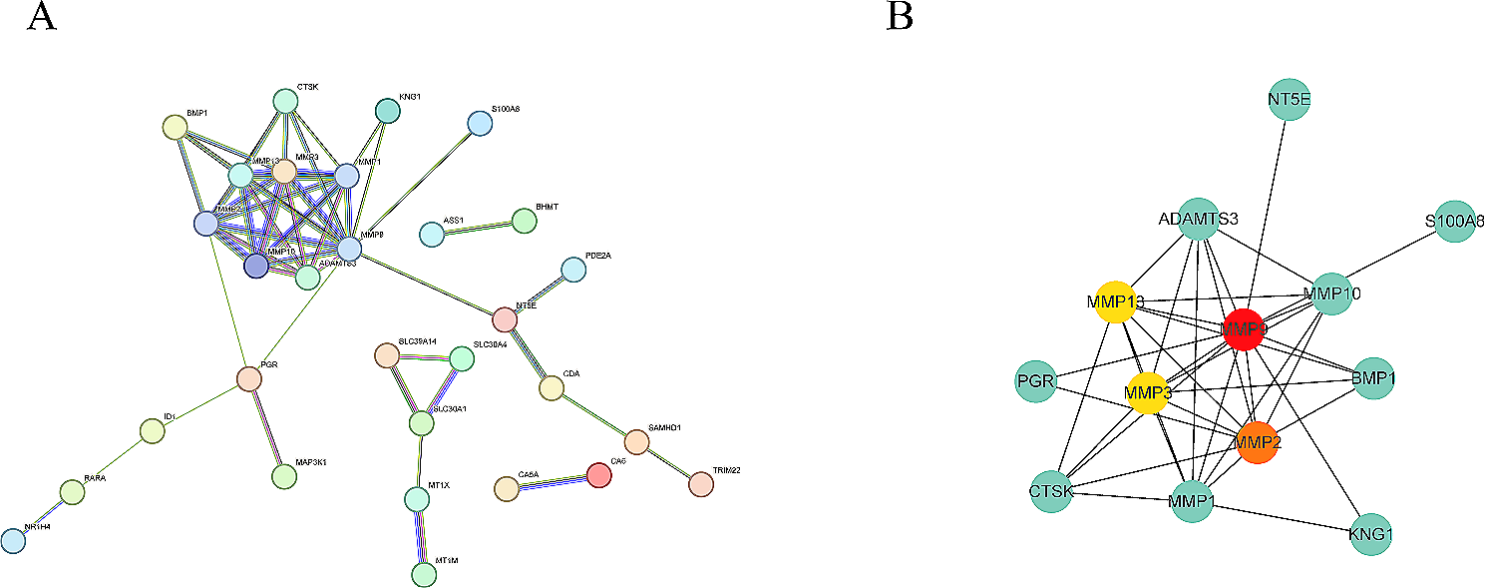
Identification of Hub genes among ZMRGs-DEGs. **(A)** PPI networks were constructed using the STRING database. **(B)** Identification of Hub genes in the PPI network using the MCC algorithm of the cytoHubba plugin in Cytoscape

We constructed a PPI network using the STRING database with the 46 ZMRGs-DEGs, resulting in a network with 46 nodes and 49 edges (Fig. 4A). Subsequently, we calculated the Hub genes in the PPI network utilizing the MCC algorithm through the cytoHubba plugin in Cytoscape, identifying MMP2, MMP3, MMP9, and MMP13 as the top 4 Hub genes (Fig. 4B).

### Validation of hub gene diagnostic performance

**Fig. 5:**
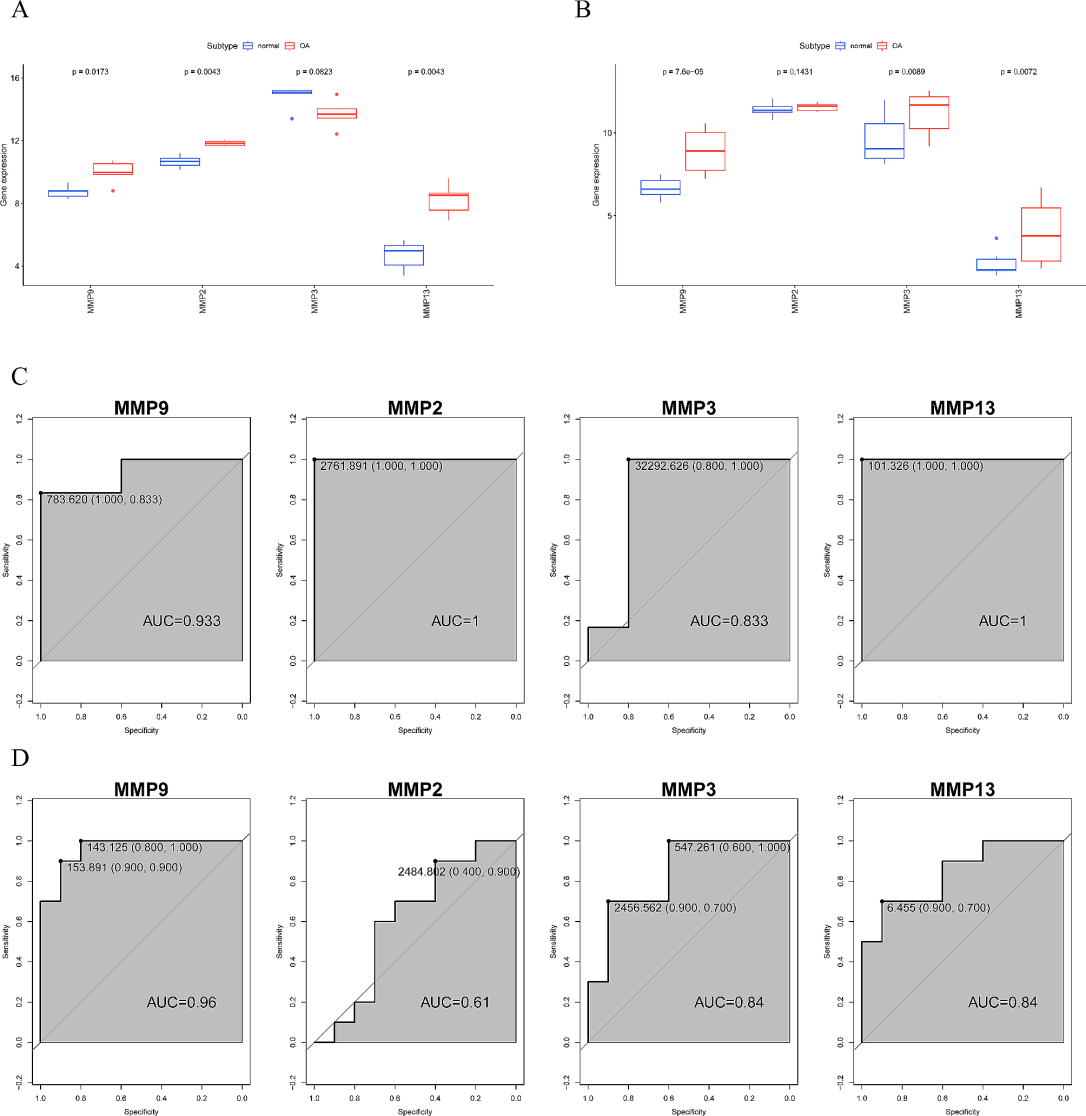
Validation of the diagnostic ability of Hub genes. **(A-B)** Expression of Hub genes in the GSE169077 training dataset (A) and GSE55235 validation dataset **(B)** between normal and OA groups. **(C-D)** Utilization of the pROC R package to draw the ROC curves of GSE169077 training set **(C)** and GSE55235 validation set **(D)** and calculate AUC

Furthermore, we conducted a comprehensive examination of the expression of Hub genes in the GSE169077 and GSE55235 datasets. Within the GSE169077 dataset, MMP9, MMP2, and MMP13 displayed notably elevated expression levels. Conversely, within the GSE55235 dataset, MMP9, MMP3, and MMP13 exhibited significantly increased expression in OA patients (Fig. 5A-B). To probe into the clinical significance of Hub genes in OA patients, we used the pROC R package to generate ROC curves for Hub genes in the GSE169077 dataset and calculated the AUCs to evaluate their predictive ability. The validation was performed using the GSE55235 dataset. The results indicated that the AUCs for MMP9, MMP2, MMP3, and MMP13 in the GSE169077 dataset were 0.933, 1, 0.833, and 1, respectively. In the validation dataset GSE55235, the AUCs were 0.96, 0.61, 0.84, and 0.84, respectively (Fig. 5C-D). In summary, Hub genes demonstrated good diagnostic performance in OA patients.

### Prediction of the gene-miRNA interaction network

**Fig. 6:**
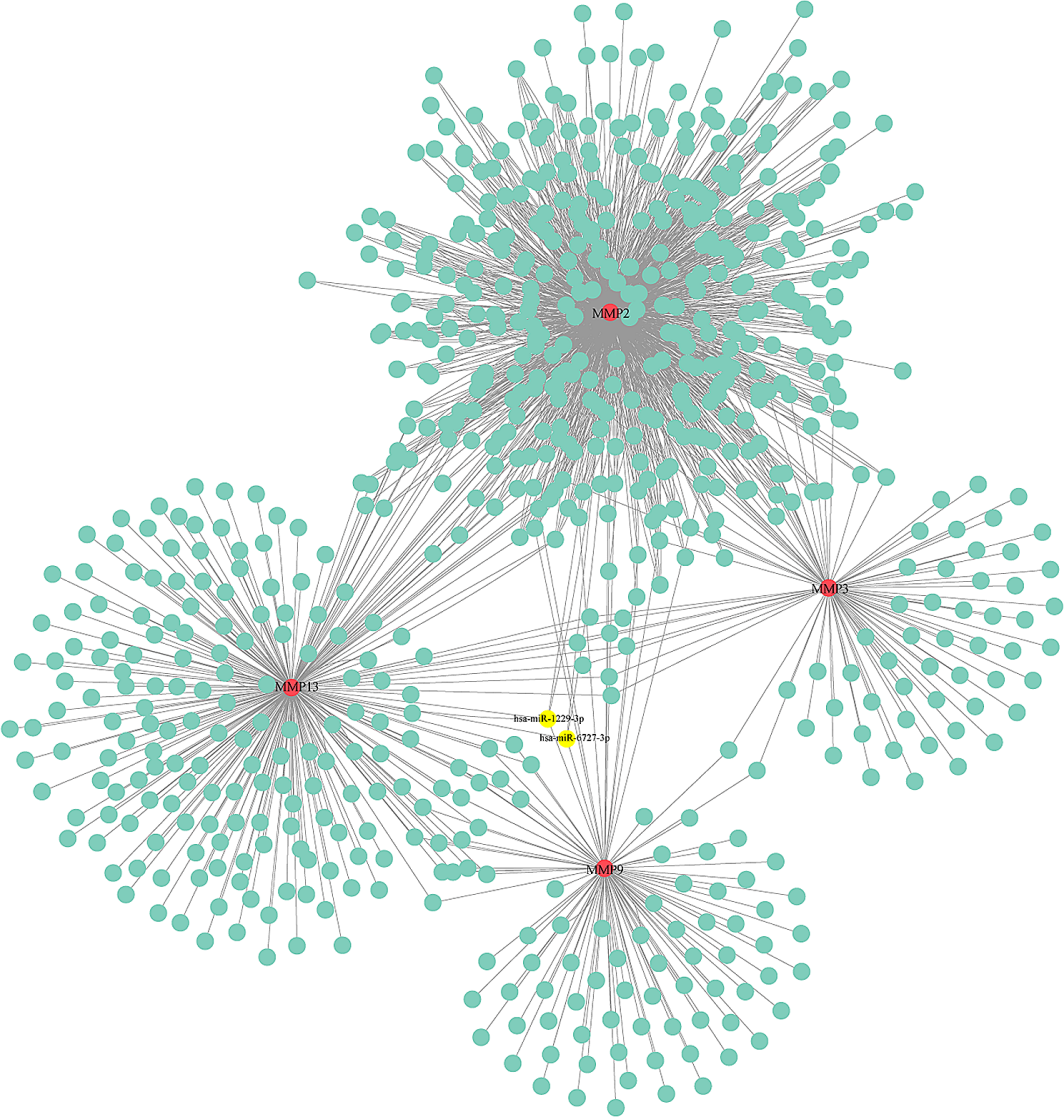
Creation of an interaction network between gene miRNAs and Hub genes using the gene-miRNA interaction network

MiRNAs play a significant role in regulating the expression levels and biological functions of protein-coding genes. We further explored the interactions between miRNAs and Hub genes. Through the gene-miRNA interaction network website, we created an interaction network between genes, miRNAs, and Hub genes, which demonstrated that hsa-miR-1229-3p and hsa-miR-6727-3p were two closely related miRNAs among the 4 Hub genes (Fig. 6).

## Discussion

OA stands as the most prevalent form of arthritis, affecting numerous patients worldwide [13]. OA exacerbates the burden on joints like the knee, accelerating cartilage damage [14]. Timely diagnosis of OA is crucial for such conditions, but current imaging-based diagnostics have significant limitations, necessitating the development of biomarkers for OA diagnosis. In addition, through the development of diagnostic techniques such as imaging, synovitis is also considered to be a common disease of OA [15]. Synovium and cartilage can also crosstalk through synovial fluid and even share some inflammatory signaling pathways [16, 17]. Therefore, two datasets GSE169077 and GSE55235 in the GEO database were selected for exploration and analysis. 841 DEGs were found between the OA samples and the healthy samples, and 46 ZMRGS-DEGs were obtained by intersecting with ZMRGs. GO enrichment results suggested that these 46 ZMRGs-DEGs primarily participate in processes related to collagen catabolic activity, extracellular matrix organization, and metallopeptidase activity. KEGG pathway analysis indicated their involvement in metabolic pathways like the IL-17 signaling pathway, Nitrogen metabolism, and the Relaxin signaling pathway. These findings suggested the active role of these genes in inflammatory processes. Therefore, we hypothesized that these genes may be crucial for OA development.

Currently, drug therapy is crucial for the treatment of OA, and the majority of patients require short-term or long-term medication. We conducted a CMap analysis to identify potential therapeutic drugs. CMap analysis identified ten small molecules, including pantoprazole, cholic-acid, etomoxir, and others, as candidate drugs, which can be explored for their respective targets. Pantoprazole is a proton pump inhibitor drug, and research has found its potential for the treatment of OA [18]. Cholic-acid belongs to bile acids, and studies have shown that activation of the bile acid receptor GPBAR1 (TGR5) can improve IL-1β-induced OA [19]. Etomoxir is a carnitine palmitoyltransferase inhibitor, and research has indicated that etomoxir can regulate mitochondrial dysfunction and activate mitochondrial autophagy to alleviate oxidative stress-induced OA [20]. While the connections between the other seven small molecules and OA are not clear, we speculate that their occurrence and progression can be controlled by these small molecules in the development of OA.

Recently, an increasing body of research has suggested that immune cell infiltration features in the onset and progression of OA [21, 22]. Studies have indicated that in OA groups, there is a significant increase in DCs, iDCs, macrophages, mast cells, APC co-inhibition, and CCR expression. In contrast, B cells, NK cells, Th2 cells, TIL, and T cells co-stimulation decrease significantly [23]. In our study, we found that in OA patients, there was a notable increase in the abundance of DCs, macrophages, mast cells, Treg cells, HLA, Parainflammation, T cell co-inhibition, T cell co-stimulation, Type I IFN Response, and Type II IFN Response. On the other hand, the levels of Tfh cells, Th1 cells, APC co-inhibition, Cytolytic activity, and MHC class I were significantly decreased in OA patients. Furthermore, the data suggested that immune checkpoint genes, including LAG3, PDCD1, and ICOS, were significantly downregulated in OA patients.

We employed the cytoHubba program in Cytoscape to examine the PPI results and identified the top 4 Hub genes, namely MMP9, MMP2, MMP3, and MMP13. These Hub genes exhibited high diagnostic efficiency in both the GSE169077 and GSE55235 datasets. All 4 of these Hub genes belong to the matrix metalloproteinase (MMP) family, which plays a role in the turnover of the extracellular matrix and the associated destruction of joint cartilage in OA [24]. Elevated expression of MMP9 has been demonstrated to contribute to the advancement of diabetes-induced OA in a rat model [25], and it has been recognized as a potential biomarker for OA in a study [26]. Studies have also demonstrated that the knockout of MMP13 in mice results in less tibial cartilage erosion compared to wild-type mice at 8 weeks post-surgery [27]. On the contrary, mice with cartilage-specific overexpression of constitutively active MMP13 have exhibited the joint pathology observed in OA [28], and targeting MMP13 has shown promise in OA therapy [29]. Research has further revealed that SOST can inhibit the post-injury expression of MMP2 and MMP3 and improve post-traumatic OA [30]. In summary, these 4 Hub genes identified here were tightly concerned with the onset and progression of OA and thus held promise as diagnostic genes for OA. Similarly, previous studies have shown that inflammatory factors produced by synovitis regulate MMPs and play an important role in OA cartilage loss [31]. Therefore, to explore upstream genes targeting Hub genes, the Gene-miRNA interaction network website creates an interaction network between Gene miRNA and Hub genes. Two miRNAs (hsa-miR-1229-3p and hsa-miR-6727-3p) that were closely related to the four Hub genes were found.

As reported, miRNA is associated with OA, and several miRNAs have been linked to the pathogenesis of the disease [32]. In this study, we established a miRNA-target gene network and identified interactions between miR-1229-3p and miR-6727-3p with the 4 Hub genes. Currently, these two miRNAs have not been extensively studied in the context of OA, but miR-1229-3p has been found to serve as a prognostic factor promoting cell viability, migration, and invasion in hepatocellular carcinoma [33].The direct role of miR-6727-3p is associated with the effect on type 2 diabetes beta cells [34]. Based on our research findings, these miRNAs may participate in the progression of OA. Nevertheless, their specific roles in OA progression require further explorations.

This work has certain limitations. Firstly, the dataset used here has a limited number of samples, which may introduce bias to the results. Secondly, more in vivo and in vitro research is needed to validate the diagnostic significance of Hub genes. Thirdly, the biological functions of Hub genes and immune microenvironment obtained in this study that can guide OS diagnosis need to be further studied. Lastly, a thorough understanding of the Hub gene targets in OA is essential for discovering and developing appropriate treatment strategies.

### Electronic supplementary material

Below is the link to the electronic supplementary material.

Supplementary Material 1: Table 1. Zinc metabolism-related genes.

Supplementary Material 2: Table 2. 30 upregulated ZMRGs-DEGs.

## Data Availability

The data and materials in the current study are available from the corresponding author on reasonable request.

## References

[1] Kraus VB, Blanco FJ, Englund M, Karsdal MA, Lohmander LS (2015). Call for standardized definitions of osteoarthritis and risk stratification for clinical trials and clinical use. Osteoarthr Cartil.

[2] Roseti L, Desando G, Cavallo C, Petretta M, Grigolo B (2019). Articular cartilage regeneration in Osteoarthritis. Cells.

[3] Nelson AE (2018). Osteoarthritis year in review 2017: clinical. Osteoarthr Cartil.

[4] Zhang Z, Huang C, Jiang Q, Zheng Y, Liu Y, Liu S, Chen Y, Mei Y, Ding C, Chen M, Gu X, Xing D, Gao M, He L, Ye Z, Wu L, Xu J, Yang P, Zhang X, Zhang Y, Chen J, Lin J, Zhao L, Li M, Yang W, Zhou Y, Jiang Q, Chu CQ, Chen Y, Zhang W, Tsai WC, Lei G, He D, Liu W, Fang Y, Wu D, Lin J, Wei CC, Lin HY, Zeng X (2020). Guidelines for the diagnosis and treatment of osteoarthritis in China (2019 edition). Ann Transl Med.

[5] Sen R, Hurley JA (2023). Osteoarthritis. *StatPearls*, StatPearls Publishing Copyright © 2023, StatPearls Publishing LLC.: Treasure Island (FL) ineligible companies.

[6] Ovesen J, Møller-Madsen B, Nielsen PT, Christensen PH, Simonsen O, Hoeck HC, Laursen MB, Thomsen JS (2009). Differences in zinc status between patients with osteoarthritis and osteoporosis. J Trace Elem Med Biology: Organ Soc Minerals Trace Elem (GMS).

[7] Yang WM, Lv JF, Wang YY, Xu YM, Lin J, Liu J, Chen JJ, Wang XZ. The daily intake levels of copper, selenium, and Zinc Are Associated with Osteoarthritis but not with rheumatoid arthritis in a cross-sectional study. Biol Trace Elem Res 2023. 10.1007/s12011-023-03636-w 36943549

[8] Zhou J, Liu C, Sun Y, Francis M, Ryu MS, Grider A, Ye K (2021). Genetically predicted circulating levels of copper and zinc are associated with osteoarthritis but not with rheumatoid arthritis. Osteoarthr Cartil.

[9] Kim JH, Jeon J, Shin M, Won Y, Lee M, Kwak JS, Lee G, Rhee J, Ryu JH, Chun CH, Chun JS (2014). Regulation of the catabolic cascade in osteoarthritis by the zinc-ZIP8-MTF1 axis. Cell.

[10] Cuffaro D, Ciccone L, Rossello A, Nuti E, Santamaria S (2022). Targeting aggrecanases for Osteoarthritis Therapy: from zinc chelation to Exosite Inhibition. J Med Chem.

[11] Chang W, Li H, Ou W, Wang SY (2023). A novel zinc metabolism-related gene signature to predict prognosis and immunotherapy response in lung adenocarcinoma. Front Immunol.

[12] Zhu K, Xiaoqiang L, Deng W, Wang G, Fu B (2021). Development and validation of a novel lipid metabolism-related gene prognostic signature and candidate drugs for patients with bladder cancer. Lipids Health Dis.

[13] Rabago D, Nourani B (2017). Prolotherapy for Osteoarthritis and Tendinopathy: a descriptive review. Curr Rheumatol Rep.

[14] Flemming DJ, Gustas-French CN (2017). Rapidly progressive osteoarthritis: a review of the clinical and radiologic presentation. Curr Rheumatol Rep.

[15] Li C, Zheng Z (2020). Identification of novel targets of knee Osteoarthritis Shared by Cartilage and Synovial tissue. Int J Mol Sci.

[16] Mathiessen A, Conaghan PG (2017). Synovitis in osteoarthritis: current understanding with therapeutic implications. Arthritis Res Ther.

[17] Wojdasiewicz P, Poniatowski LA, Szukiewicz D (2014). The role of inflammatory and anti-inflammatory cytokines in the pathogenesis of osteoarthritis. Mediators Inflamm.

[18] Bianchi Porro G, Lazzaroni M, Imbesi V, Montrone F, Santagada T (2000). Efficacy of pantoprazole in the prevention of peptic ulcers, induced by non-steroidal anti-inflammatory drugs: a prospective, placebo-controlled, double-blind, parallel-group study. Dig Liver Disease: Official J Italian Soc Gastroenterol Italian Association Study Liver.

[19] Huang H, Lei H, Yang F, Fan X, Dang Q, Li Y (2018). Activation of the bile acid receptor GPBAR1 (TGR5) ameliorates interleukin-1β (IL-1β)- induced chondrocytes senescence. Biomed Pharmacother.

[20] Jiang N, Xing B, Peng R, Shang J, Wu B, Xiao P, Lin S, Xu X, Lu H (2022). Inhibition of Cpt1a alleviates oxidative stress-induced chondrocyte senescence via regulating mitochondrial dysfunction and activating mitophagy. Mech Ageing Dev.

[21] Woodell-May JE, Sommerfeld SD (2020). Role of inflammation and the Immune System in the progression of Osteoarthritis. J Orthop Research: Official Publication Orthop Res Soc.

[22] Miller RJ, Malfait AM, Miller RE (2020). The innate immune response as a mediator of osteoarthritis pain. Osteoarthr Cartil.

[23] Wang W, Ou Z, Peng J, Wang N, Zhou Y (2022). Bioinformatics-based analysis of potential candidates chromatin regulators for immune infiltration in osteoarthritis. BMC Musculoskelet Disord.

[24] Rowan AD, Litherland GJ, Hui W, Milner JM (2008). Metalloproteases as potential therapeutic targets in arthritis treatment. Expert Opin Ther Targets.

[25] Luo S, Li W, Wu W, Shi Q (2021). Elevated expression of MMP8 and MMP9 contributes to diabetic osteoarthritis progression in a rat model. J Orthop Surg Res.

[26] Li S, Wang H, Zhang Y, Qiao R, Xia P, Kong Z, Zhao H, Yin L (2021). COL3A1 and MMP9 serve as potential diagnostic biomarkers of Osteoarthritis and are Associated with Immune Cell Infiltration. Front Genet.

[27] Little CB, Barai A, Burkhardt D, Smith SM, Fosang AJ, Werb Z, Shah M, Thompson EW (2009). Matrix metalloproteinase 13-deficient mice are resistant to osteoarthritic cartilage erosion but not chondrocyte hypertrophy or osteophyte development. Arthritis Rheum.

[28] Neuhold LA, Killar L, Zhao W, Sung ML, Warner L, Kulik J, Turner J, Wu W, Billinghurst C, Meijers T, Poole AR, Babij P, DeGennaro LJ (2001). Postnatal expression in hyaline cartilage of constitutively active human collagenase-3 (MMP-13) induces osteoarthritis in mice. J Clin Invest.

[29] Hu Q, Ecker M. Overview of MMP-13 as a Promising Target for the treatment of Osteoarthritis. Int J Mol Sci 2021, *22* (4). 10.3390/ijms22041742 PMC7916132 33572320

[30] Chang JC, Christiansen BA, Murugesh DK, Sebastian A, Hum NR, Collette NM, Hatsell S, Economides AN, Blanchette CD, Loots GG (2018). SOST/Sclerostin Improves Posttraumatic Osteoarthritis and inhibits MMP2/3 expression after Injury. J bone Mineral Research: Official J Am Soc Bone Mineral Res.

[31] Jarecki J, Malecka-Masalska T, Kosior-Jarecka E, Widuchowski W, Krasowski P, Gutbier M, Dobrzynski M, Blicharski T. Concentration of selected metalloproteinases and osteocalcin in the serum and synovial fluid of obese women with advanced knee osteoarthritis. Int J Environ Res Public Health 2022, *19* (6). 10.3390/ijerph19063530 PMC8953048 35329213

[32] Zhang QC, Hu SQ, Hu AN, Zhang TW, Jiang LB, Li XL (2021). Autophagy-activated nucleus pulposus cells deliver exosomal miR-27a to prevent extracellular matrix degradation by targeting MMP-13. J Orthop Research: Official Publication Orthop Res Soc.

[33] Zhang C, Zhang Q, Li H, Wu Y (2021). Mir-1229-3p as a prognostic predictor facilitates cell viability, Migration, and Invasion of Hepatocellular Carcinoma. Hormone Metabolic Res = Hormon- und Stoffwechselforschung = Horm et Metab.

[34] Zraika S, Barrow B, Enquobahrie D, Bammler TK, Macdonald J, Srinouanprachanh S, Chuen Gary Chan K, Boyko EJ, Kahn SE, Wander P. 1765-P: direct ß-Cell effects of miR-6727-3p, an miRNA related to improved ß-Cell function in humans. Diabetes 2023, *72* (Supplement_1).

